# Out-of-Hospital Cardiac Arrest in US Airports

**DOI:** 10.1001/jamanetworkopen.2025.29754

**Published:** 2025-08-29

**Authors:** Aditya C. Shekhar, Joshua Kimbrell, Timothy Friedmann, Jacob Stebel, Bojana Milekic, Ryan Huebinger, Ryan Coute, Ethan E. Abbott, Keith J. Ruskin, Benjamin S. Abella

**Affiliations:** 1 Icahn School of Medicine at Mount Sinai, New York City, New York; 2 Department of Prehospital Care, MediSys Health Network, New York, New York; 3 Department of Emergency Medicine, Mount Sinai Hospital, New York, New York; 4 Institute for Critical Care Medicine, Mount Sinai Hospital, New York, New York; 5 Department of Emergency Medicine, University of New Mexico, Albuquerque; 6 Department of Emergency Medicine, University of Alabama, Birmingham; 7 Department of Anaesthesia and Critical Care, University of Chicago, Chicago, Illinois

## Abstract

**Importance:**

Airports are uniquely situated to facilitate several aspects of the out-of-hospital cardiac arrest (OHCA) chain of survival, including a high likelihood of cardiac arrest events being witnessed and the ready availability of automated external defibrillators (AEDs). Focused analyses suggest airports are associated with a higher prevalence of witnessed cardiac arrest and AED use; however, there is a lack of national US data on the topic to facilitate comparison of response characteristics with other OHCA events occurring in nonairport locations.

**Objective:**

To compare OHCAs occurring at airports with OHCAs occurring in nonairport, nonresidential settings.

**Design, Setting, and Participants:**

This cross-sectional study used a national-level database of emergency medical services (EMS) activations in the US, the National Emergency Medical Services Information System. Inclusion criteria were any adult (≥18 years) experiencing cardiac arrest prior to EMS arrival between January 1, 2022, and December 31, 2023.

**Main Outcomes and Measures:**

Key cardiac arrest variables (witnessed status, pre-EMS cardiopulmonary resuscitation [CPR] or AED use, shockable rhythms, and etiology) were compared across cardiac arrests in airports and nonairport, nonresidential settings.

**Results:**

A total of 1194 OHCAs in airport settings (452 among individuals aged 18-60 years [37.9%]; 867 among men [72.6%]) and 312 306 OHCAs in nonairport, nonresidential settings (147 431 among individuals aged 18-60 years [47.2%]; 211 364 among men [67.7%]) met inclusion criteria. Airports were associated with significantly higher rates of nontraumatic etiologies (96.1% [1148 of 1194] vs 89.5% [279 462 of 312 306]; *P* < .001), witnessed cardiac arrests (81.0% [967 of 1194] vs 43.3% [135 091 of 312 306]; *P* < .001), CPR before EMS arrival (62.6% [742 of 1194] vs 47.8% [149 166 of 312 306]; *P* < .001), AED use before EMS arrival (56.3% [672 of 1194] vs 32.0% [100 020 of 312 306]; *P* < .001), shockable rhythms (28.6% [341 of 1194] vs 13.8% [43 187 of 312 306]; *P* < .001), and return of spontaneous circulation (40.7% [486 of 1194] vs 23.8% [74 467 of 312 306]; *P* < .001) compared with nonairport, nonresidential settings.

**Conclusion and Relevance:**

This study suggest that airports are associated with substantially improved OHCA response and survival outcomes compared with other nonresidential settings. Further efforts are needed to establish strategies to translate the relevant parts of the chain of OHCA survival found at airports to other public venues.

## Key Points

**Question:**

How are out-of-hospital cardiac arrest (OHCA) cases at US airports different from OHCA cases at other public venues?

**Findings:**

This cross-sectional study used a nationwide database of emergency medical services (EMS) activations in the US to compare OHCA in airports with nonairport nonresidential settings. Airports were associated with significantly increased rates of witnessed cardiac arrests, cardiopulmonary resuscitation and automated external defibrillator use before EMS arrival, shockable rhythms, and return of spontaneous circulation.

**Meaning:**

This study suggests that, due to a variety of factors, airports are associated with improved OHCA response and survival outcomes compared with other nonresidential settings—other public venues should try to replicate these factors to improve their cardiac arrest preparedness.

## Teaser

This cross-sectional study uses data from the the National Emergency Medical Services Information System to compare out-of-hospital cardiac arrests occurring at airports with out-of-hospital cardiac arrests occurring in nonairport, nonresidential settings.

## Introduction

Airports have several unique features that facilitate out-of-hospital cardiac arrest (OHCA) survival.^1^ The open design of airport terminals and the large volume of passengers increase the likelihood of any cardiac arrest being witnessed.^2^ Pervasive video surveillance allows prompt activation of emergency response resources even if someone is not physically present at the cardiac arrest location. Airports also have been associated with high rates of bystander cardiopulmonary resuscitation (CPR) and automated external defibrillator (AED) use.^3^ In addition, airports are staffed with police officers, security guards, and other personnel who are likely trained in first aid and can rapidly respond to cardiac arrests. Automated external defibrillators in airport terminals are also readily accessible and highlighted by visible permanent signage. Large airports also tend to have dedicated on-site EMS, allowing for quick response times.

Likely due to some combination of these factors, airports have historically exhibited high OHCA survival rates. Chatterjee et al^4^ analyzed OHCA outcomes at Seattle-Tacoma International Airport between January 2004 and December 2019. Across 109 cardiac arrests, 89% were witnessed, 78% received CPR, 55% had an AED applied prior to EMS arrival, 72% were initially shockable, 68% achieved return of spontaneous circulation (ROSC), and 44% had survival to hospital discharge. Similarly, Gantzel Nielsen et al^5^ examined cardiac arrest at Copenhagen International Airport between May 2015 and May 2019 and found that, across 23 cardiac arrests, 74% received bystander CPR, 44% received bystander defibrillation, 78% achieved ROSC, and 57% survived to hospital discharge.

Despite promising single-center evidence, relatively little multicenter research has specifically examined airport cardiac arrests at broader levels. The specific factors underpinning high survival rates in airports (eg, the relative contribution of cardiac arrest witness status vs AED availability that is relatively unique to airports) remain an important knowledge gap. In this study, we used a large and nationally representative database of EMS activations in the US to specifically compare OHCAs occurring at airports with OHCAs occurring in other nonresidential settings.

## Methods

### Data Source

The National Emergency Medical Services Information System (NEMSIS) is a database containing more than 100 million EMS activations from throughout the US, representing more than 87% of all EMS activations nationwide.^6^ The database is populated directly from patient care reports produced by EMS crews during routine patient encounters.^7^ The Icahn School of Medicine at Mount Sinai Institutional Review Board previously deemed research using the NEMSIS database exempt from review and waived the need for informed consent owing to the use of retrospective data obtained via routine clinical care. This cross-sectional study followed the Strengthening the Reporting of Observational Studies in Epidemiology (STROBE) reporting guideline.

### Study Population and Variables

**Figure 1.**
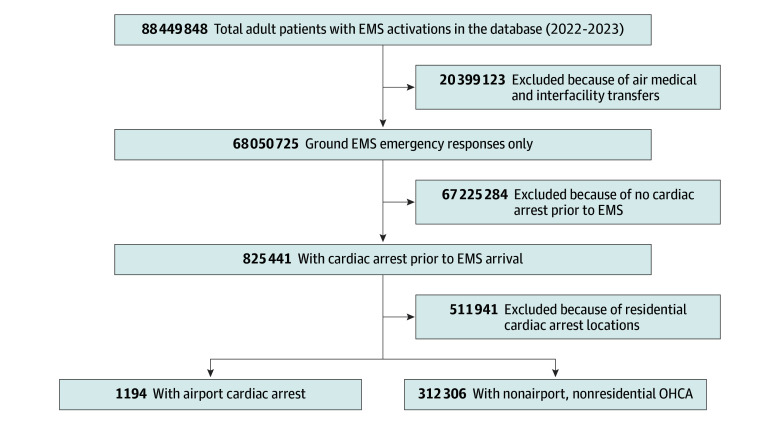
Derivation of Study Population, Including Airport vs Nonairport, Nonresidential Out-of-Hospital Cardiac Arrest (OHCA) EMS indicates emergency medical services.

We examined all OHCA events among adults (aged ≥18 years) between January 1, 2022, and December 31, 2023, with a response from ground-based EMS crews. Cardiac arrests occurring after EMS arrival, involving air medical resources, or involving interfacility transfers were excluded (Figure 1). In addition to basic demographic information, specific cardiac arrest variables of interest included (1) witnessed status, (2) CPR prior to EMS arrival, (3) AED use prior to EMS arrival, (4) first monitored cardiac arrest rhythm, (5) etiology of cardiac arrest, and (6) ROSC achievement.

### Statistical Analysis

The data were queried through a proprietary, web-based workspace specific to NEMSIS. From there, data outputs were copied into a Google spreadsheet, where statistical tests were run. Cardiac arrests were stratified by the arrest location documented by EMS—specifically, airport vs nonairport nonresidential settings. To build a cohort of nonairport, nonresidential settings as the comparator group, we examined all cardiac arrests that took place at documented locations other than airports. From there, we subsequently excluded all incident location types with at least 500 cardiac arrests documented as being residential. This method was chosen because there are more than 200 incident location types where at least 1 cardiac arrest took place. Doing this resulted in the elimination of approximately 500 000 cardiac arrests taking place within residential settings. Comparisons between cardiac arrests occurring at airports vs nonairport, nonresidential settings were made across the aforementioned cardiac arrest variables. Two-proportion, 2-tailed *z* score tests were used with statistical significance defined as *P* < .05.

## Results

**Figure 2.**
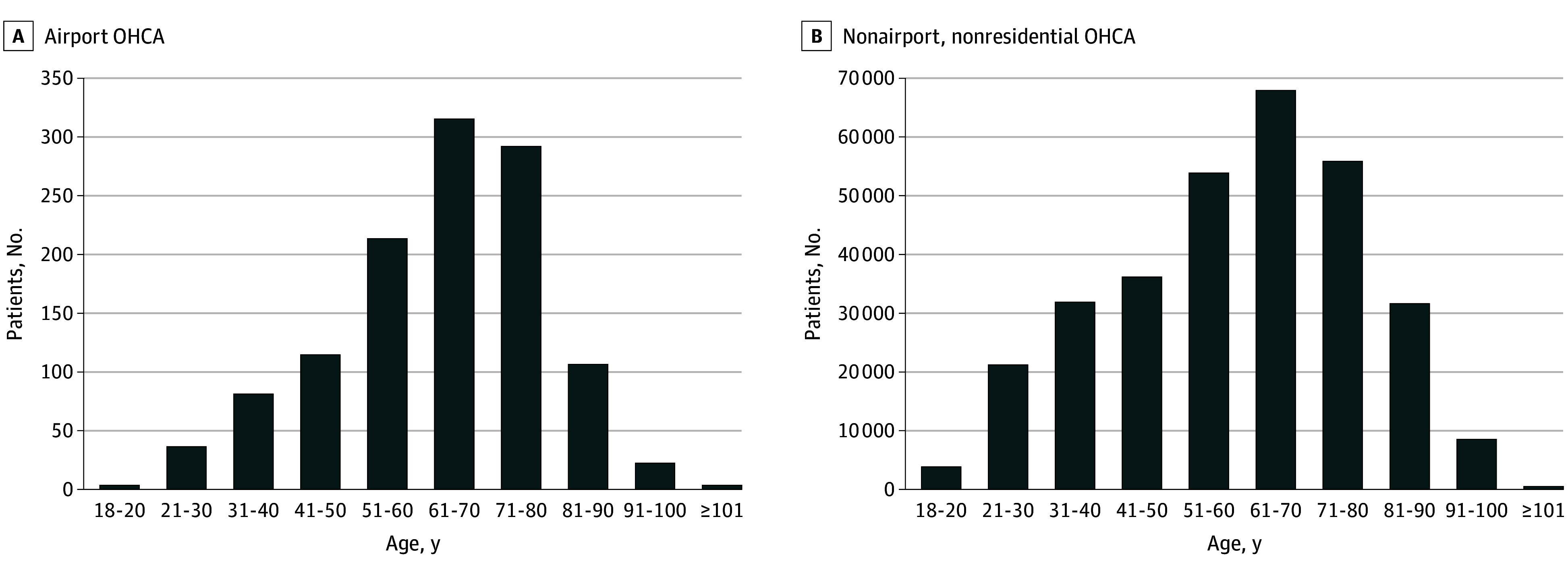
Patient Age for Airport and Nonairport, Nonresidential Out-of-Hospital Cardiac Arrest (OHCA) Age distribution of OHCA cases across airport (A) vs nonairport, nonresidential (B) locations.

A total of 1194 OHCAs in airport settings (452 among individuals aged 18-60 years [37.9%]; 867 among men [72.6%] and 324 among women [27.1%]) and 312 306 OHCAs in nonairport nonresidential settings (147 431 among individuals aged 18-60 years [47.2%]; 211 364 among men [67.7%] and 99 365 among women [31.8%]) met inclusion criteria (Table). Regarding demographics, OHCAs in airports were significantly more likely than OHCAs at nonairport, nonresidential settings to occur among men (72.6% [867 of 1194] vs 67.7% [211 364 of 312 306]; *P* < .001) but significantly less likely to involve patients between 18 and 60 years of age (37.9% [452 of 1194] vs 47.2% [147 431 of 312 306]; *P* < .001) (Figure 2). Airport OHCAs were significantly more likely than OHCAs at nonairport, nonresidential settings to be witnessed (81.0% [967 of 1194] vs 43.3% [135 091 of 312 306]; *P* < .001) and more likely to be associated with nontraumatic etiologies (96.1% [1148 of 1194] vs 89.5% [279 462 of 312 306]; *P* < .001). Airport OHCAs were also more likely than OHCAs at nonairport, nonresidential settings to be associated with CPR prior to EMS arrival (62.6% [742 of 1194] vs 47.8% [149 166 of 312 306]; *P* < .001) and AED use prior to EMS arrival (56.3% [672 of 1194] vs 32.0% [100 020 of 312 306]; *P* < .001). Finally, airport OHCAs were more likely than OHCAs at nonairport, nonresidential settings to exhibit shockable initial cardiac rhythms (28.6% [341 of 1194] vs 13.8% [43 187 of 312 306]; *P* < .001) and frequently achieved ROSC (40.7% [486 of 1194] vs 23.8% [74 467 of 312 306]; *P* < .001).

**Table.** Cardiac Arrest Chains of Survival at US Airports vs Nonairport, Nonresidential Locations

Cardiac arrest parameter	Location, No. (%)		*z* Score	*P* value
	Airport (n = 1194)	Nonairport, nonresidential (n = 312 306)		
Male	867 (72.6)	211 364 (67.7)	3.6393	<.001
Witnessed cardiac arrest	967 (81.0)	135 091 (43.3)	26.2563	<.001
Aged 18-60 y	452 (37.9)	147 431 (47.2)	−6.4606	<.001
Nontraumatic cardiac arrest	1148 (96.1)	279 462 (89.5)	7.500	<.001
Pre-EMS CPR	748 (62.6)	149 166 (47.8)	10.2762	<.001
Pre-EMS AED use	672 (56.3)	100 020 (32.0)	17.9153	<.001
Initially shockable rhythm	341 (28.6)	43 187 (13.8)	14.6927	<.001
ROSC	486 (40.7)	74 467 (23.8)	13.6323	<.001

Abbreviations: AED, automated external defibrillator; CPR, cardiopulmonary resuscitation; EMS, emergency medical services; ROSC, return of spontaneous circulation.

## Discussion

Our analysis of an extensive, nationally representative database of EMS activations in the US found that airports are associated with several improvements in key components of the chain of OHCA survival compared with nonairport nonresidential settings. Our findings are consistent with several smaller-scale studies.^4,5^ First, we show that airport cardiac arrests were almost twice as likely to be witnessed than nonairport, nonresidential cardiac arrests. This finding is unsurprising given that the layouts of most airport terminals are designed to congregate large groups of people together in collective spaces. A person in an airport is therefore more likely to experience a cardiac arrest in close proximity to others. Moreover, airport terminals are under a high degree of surveillance, which can help in the recognition of a cardiac arrest even if the individual experiencing cardiac arrest is alone.

Second, airport cardiac arrests were significantly more likely to be associated with CPR and AED use prior to EMS arrival. Airports are often staffed with ample personnel (eg, public safety officers) trained in CPR who can render care—alongside willing bystanders—prior to the arrival of EMS. High rates of AED use prior to EMS arrival likely reflect airports featuring a high density of AEDs over a given area. Furthermore, AEDs in airports are often made highly visible with signage to make travelers and staff aware of their location—ongoing research is being conducted to optimize AED sign design and placement.^8,9,10,11^ Use of an AED by bystanders before EMS arrival has been associated with improved OHCA survival and functional outcomes.^12^ A high density of AEDs also means that an AED is relatively close to any given cardiac arrest and can be easily accessed by rescuers. This is important because individuals experiencing cardiac arrest are most likely to receive shockable rhythms if an AED is located in the immediate vicinity.^13^ Similarly, the higher rates of shockable arrest rhythms for airport cardiac arrests might reflect that care is being rendered earlier and/or that cardiac monitors are being applied more rapidly. Many patients with cardiac arrest found to have nonshockable rhythms may have originally experienced cardiac arrest with shockable rhythms that deteriorated to a nonshockable rhythm before monitoring was applied. Conversely, bystander CPR has been shown to prolong the duration of shockable rhythms.^14,15^

Finally, we showed that airport cardiac arrests were associated with higher rates of ROSC achievement compared with cardiac arrests in nonairport, nonresidential settings. This finding likely reflects all of the aforementioned factors, including higher rates of witnessed arrests and pre-EMS interventions. Individuals experiencing cardiac arrest at many large airports also benefit from the presence of on-site dedicated EMS staff members who may be able to begin administering care rapidly after onset of cardiac arrest.^1,16^ Our findings present an opportunity to improve cardiac arrest survival globally; airport designs may provide a model for public spaces that can facilitate the cardiac arrest chain of survival promptly and reliably.

### Limitations

Our study is associated with several limitations. First, although the use of a large database allows for the construction of a multicenter cohort, we lack data on long-term outcomes (eg, survival to admission or neurologic status) because national-level EMS data cannot easily link with hospital data. As such, we were forced to use ROSC achievement as the closest surrogate end point for the ultimate outcome. Second, we did not have any data on pre–cardiac arrest comorbidities, which may affect the likelihood of successful resuscitation. Thus, some of the difference in ROSC achievement might be explained by differences between the cohorts that could not be controlled for. Third, we built the airport cohort based on EMS documentation. It is possible some cardiac arrest locations may have been misclassified, and we could not further subclassify the airport location (eg, the specific location within the airport). Despite these limitations, our study represents the largest comparison of airport cardiac arrests and provides key insight into airports’ contribution to aspects of the cardiac arrest chain of survival.

## Conclusions

Our cross-sectional study of a broad and nationally representative database of EMS activations in the US found that airports are associated with dramatic improvements in key components of the OHCA chain of survival. Further study is needed to find strategies to translate the chain of survival found at airports to other public venues.

## Notes

Supplement.
Data Sharing Statement
