# Supplementary material for: Out-of-Hospital Cardiac Arrest in US Airports
Source: JAMA Netw Open. 2025 Aug 29;8(8):e2529754. doi: 10.1001/jamanetworkopen.2025.29754 (PMC12397889; doi:10.1001/jamanetworkopen.2025.29754)
Supplement: Supplement. — Data Sharing Statement [file jamanetwopen-e2529754-s001.pdf]

Data Sharing Statement

Shekhar. Out-of-Hospital Cardiac Arrest in US Airports. *JAMA Netw Open*. Published August 29, 2025. doi:10.1001/jamanetworkopen.2025.29754

Data
Data available: No
